# Iron-overload-induced ferroptosis in mouse cerebral toxoplasmosis promotes brain injury and could be inhibited by Deferiprone

**DOI:** 10.1371/journal.pntd.0011607

**Published:** 2023-08-31

**Authors:** Chong Wang, Linding Xie, Yien Xing, Min Liu, Jun Yang, Nannan Gao, Yihong Cai

**Affiliations:** 1 Department of Health Inspection and Quarantine, School of Public Health, Anhui Medical University, Hefei, China; 2 Anhui Provincial Laboratory of Microbiology and Parasitology, and Anhui Provincial Laboratory of Zoonoses of High Institutions, Anhui Medical University, Hefei, China; Ben-Gurion University of the Negev, ISRAEL

## Abstract

Iron is a trace metal element that is essential for the survival of cells and parasites. The role of iron in cerebral toxoplasmosis (CT) is still unclear. Deferiprone (DFP) is the orally active iron chelator that binds iron in a molar ratio of 3:1 (ligand:iron) and promotes urinary iron excretion to remove excess iron from the body. The aims of this experiment were to observe the alterations in iron in brains with *Toxoplasma gondii* (*T*. *gondii*) acute infections and to investigate the mechanism of ferroptosis in CT using DFP. We established a cerebral toxoplasmosis model *in vivo* using TgCtwh3, the dominant strains of which are prevalent in China, and treated the mice with DFP at a dose of 75 mg/kg/d. Meanwhile, we treated the HT-22 cells with 100 μM DFP for half an hour and then infected cells with TgCtwh3 *in vitro*. A qRT-PCR assay of *Tg*SAG1 levels showed a response to the *T*. *gondii* burden. We used inductively coupled plasma mass spectrometry, an iron ion assay kit, Western blot analysis, glutathione and glutathione disulfide assay kits, a malonaldehyde assay kit, and immunofluorescence to detect the ferroptosis-related indexes in the mouse hippocampus and HT-22 cells. The inflammatory factors interferon-γ, tumor necrosis factor-α, transforming growth factor-β, and arginase 1 in the hippocampus and cells were detected using the Western blot assay. Hematoxylin and eosin staining, electron microscopy, and the Morris water maze experiment were used to evaluate the brain injuries of the mice. The results showed that TgCtwh3 infection is followed by the activation of ferroptosis-related signaling pathways and hippocampal pathological damage in mice. The use of DFP led to ferroptosis resistance and attenuated pathological changes, inflammatory reactions and *T*. *gondii* burden of the mice, prolonging their survival time. The HT-22 cells with TgCtwh3 activated the ferroptosis pathway and was inhibit by DFP *in vitro*. In TgCtwh3-infected cells, inflammatory response and mitochondrial damage were severe, but these effects could be reduced by DFP. Our study elucidates the mechanism by which *T*. *gondii* interferes with the host’s iron metabolism and activates ferroptosis, complementing the pathogenic mechanism of CT and further demonstrating the potential value of DFP for the treatment of CT.

## Author summary

Iron metabolism is closely linked to host’s immunity and is involved in inflammatory and infectious processes. Iron is an important target for the host in its fight against pathogens. Meanwhile, iron is likewise a nutritional factor of pathogens and is closely associated to the survival and reproduction of *T*. *gondii*. The iron acquisition mechanisms of *T*. *gondii* may interfere with or disrupt host iron homeostasis and may induce ferroptosis in the host. Cerebral toxoplasmosis (CT) is a life-threatening infection caused by *T*. *gondii*. A comprehensive understanding of the iron metabolism status of hippocampal cells in the cerebral toxoplasmosis mouse model will help to understand the *T*. *gondii* iron acquisition pattern. To provide clues for subsequent studies on the specific mechanisms by which *T*. *gondii* regulates host iron metabolism and the development of relevant anti-Toxoplasma drugs. Here, our results showed that ferroptosis and inflammation appeared in the hippocampus after *T*. *gondii* infection. The DFP inhibits ferroptosis and attenuates inflammation, improving overall health and hippocampal pathological manifestations in infected mice. Our findings highlight that maintaining iron homeostasis and targeting ferroptosis may be a potential therapeutic approach for toxoplasmosis.

## Introduction

*Toxoplasma gondii* (*T*. *gondii*) is an intracellular parasitic protozoan that can invade all nucleated cells; it has a wide range of intermediate hosts and is highly prevalent worldwide [1]. Cerebral toxoplasmosis (CT) is one of the main toxoplasmosis caused by *T*. *gondii*. The existing drug therapies cannot completely eliminate *T*. *gondii* in the host. *T*. *gondii* is often latent in the central nervous system (CNS), leading to chronic infection. While in immunocompromised or suppressed patients, the reactivation of *T*. *gondii* cysts in the CNS can lead to severe tissue destruction and life-threatening Toxoplasma encephalitis (TE). In short, toxoplasmosis is associated with a very high disease burden around the world [2]. The rise of the Acquired Immune Deficiency Syndrome epidemic and the increase in the number of chemotherapy or transplant recipients has also drawn attention to chronic *T*. *gondii* infections that are latent in the CNS and, the reactivation of which causes TE [3,4]. *T*. *gondii* infection can lead to inflammation and oxidative stress and may have a number of deleterious consequences for host cells, even death [5]. One study showed that the type I strain of *T*. *gondii* was mostly a strong strain that could cause 100% mortality in infected mice, with most mice dying in the acute phase (LD100 = 1); the type II strain of *T*. *gondii* was less virulent in mice (LD50 ≥ 10^3^) and easily formed encapsulation in the brains, skeletal muscles, and other tissues, causing invisible infections; meanwhile, type III was a non-virulent strain (LD50 ≥ 10^5^) [6]. In China, the *T*. *gondii* Chinese Strain I (Toxo DB#9) is the main prevalent strain [7]. TgCtwh3 is a representative strain of Chinese Strain I, which is also the strain used in this study. The 10^3^ tachyzoites of TgCtwh3 eventually lead to the death of all inoculated mice, and can be considered to be equally as virulent as the type I RH strain in mice [8,9]. Based on the results of the next-generation sequencing analysis of Chinese I strain, it might have unique pathogenicity and immune response processes to hosts including humans [10].

Iron is involved in a variety of physiological and biochemical reactions in cells, such as energy metabolism, neurotransmitter metabolism, myelin formation, and cell proliferation [11,12]. It is an essential nutrient for growth and development, as well as the function of the human CNS. Iron nutrient in infants and young children can adversely affect their mental, motor, cognitive and intellectual abilities [13–15]. Iron overload is also harmful, as iron is involved in the production of reactive oxygen species, and excess iron can lead to oxidative stress and neurodegeneration in the CNS [16]. Iron accumulation in the central nervous system is thought to be one of the causative mechanisms of several degenerative diseases and other brain disorders [17,18]. Therefore, iron homeostasis is necessary for the proper functioning of the CNS. Acquisition of iron from the host is necessary for pathogens’ survival and reproduction, and pathogens can compete for iron with host cells in a variety of ways [19]. The process of host cells competing with pathogens for iron may lead to an imbalance in their own iron homeostasis. Iron is also an essential nutrient for the survival of *T*. *gondii*, and it has been reported that *T*. *gondii* infection can lead to increased tissue and serum iron levels in the small intestine, lung, and liver [20]. In addition, *T*. *gondii* infection has been shown to induce enhancement in transferrin receptor 1 mRNA expression levels in trophoblastic cells. Deferoxamine (DFO, an iron chelator) decreased the parasite’s multiplication in both trophoblastic cells [21]. In summary, *T*. *gondii* interferes with the iron homeostasis of host cells; this may be one of the pathogenic mechanisms of toxoplasmosis.

Ferroptosis is a form of cell death in which iron homeostasis is imbalanced and iron-dependent lipid hydroperoxides accumulate to lethal levels; this involves multiple biological processes such as iron metabolism and oxidative stress [22]. Ferroptosis usually plays a pro-inflammatory role in the disease process [23]. Deferiprone (DFP) is an iron-chelating agent that can penetrate the blood–brain barrier [24]. It exhibits ferroptosis-inhibiting activities and anti-inflammatory effects and has been used as a potential drug in clinical trials for the treatment of neurodegenerative disease [25]. Iron accumulation increases susceptibility to ferroptosis. An important way to inhibit ferroptosis in mice neurons is to use DFP, which can cross the blood–brain barrier, penetrate cells, and reduce excess iron accumulation. DFP ameliorates disease phenotypes in Tau mouse models [26] and general anesthesia-induced neurotoxicity in rodents [27]. The ferroptosis pathway may contribute to the demyelination process during the inflammatory phase of a lysolecithin (LPC)-induced focal demyelination model in C57BL/6J mice, and seven days of intraperitoneal treatment with DFP reduced microgliosis and inflammation and prevented axonal damage and retinal ganglion cell loss [28]. However, to our knowledge, the effect of DFP treatment on ferroptosis in CT has not been reported.

*T*. *gondii* infection could regulate the host cellular trace element metabolism in mice [29]. The ferroptosis of CT may potentially occur under iron overload conditions. Elucidating the roles of iron overload in the ferroptosis of CT and the underlying mechanisms would contribute to the development of novel therapeutic strategies for CT. We thus conducted the current study to determine whether iron overload could lead to ferroptosis in CT and explored whether inhibiting ferroptosis in CT could alleviate brain injuries *in vivo* and *in vitro*.

## Materials and methods

### Ethics statement

All experimental procedures were approved by Animal Experimental Ethics Committee of Anhui Medical University, with permit number 20211187, and performed according to the Guidelines for the Care and Use of Research Animals established by the university. All animals were housed in rooms with temperature and humidity control in the appropriate range, 12-hour light/dark cycle, and free access to food and filtered water in standard cages.

### Parasites

The Chinese 1 dominant genotype TgCtwh3 strain of *T*. *gondii* (Toxo DB#9) was used to infect the animals in this study. TgCtwh3 strain (a strong virulent strain) was obtained from Anhui Provincial Key Laboratory of Microbiology & Parasitology, Anhui Medical University, and was conserved through mouse passages. Seven days after inoculation, tachyzoites were harvested from the abdominal cavity and used to infect experimental animals.

### Animals

C57BL/6 female mice, 7–8 weeks old, were purchased from Hangzhou Ziyuan Experimental Animal Technology Co., Ltd (production license number: SCXK2019-0004) and acclimatized for one week for subsequent experiments. Non-infected Animals given vehicle (sterile 0.9% saline) for 7 consecutive days were analyzed as con + vehicle group. Non-infected Animals given DFP (3-hydroxy-1,2-dimethyl-4(1H)-pyridone, 75 mg/kg [30,31], dissolved in sterile 0.9% saline, gavage, MCE, United States) for 7 consecutive days were analyzed as con + DFP group. Infection models of *T*. *gondii* in C57BL/6 mice via intraperitoneal inoculation with 1 × 10^3^ tachyzoites [29] of TgCtwh3 suspended in 200 μL of 0.9% saline. Infected Animals given vehicle (sterile 0.9% saline) for 7 consecutive days were the TgCtwh3 + vehicle group. Infected animals given with DFP (75 mg/kg, dissolved in sterile 0.9% saline, gavage) for 7 consecutive days after 0.5 h post-infection were treated as the TgCtwh3 + DFP group [32]. The mice were anesthetized with 10% pentobarbital before sacrifice, and the serum and hippocampal tissues were collected and snap-frozen in liquid nitrogen for analysis.

### Detection of *T*. *gondii* ITS-1 gene by PCR

DNA extraction was performed from the hippocampus of mice using the DNA Extraction Kit (AG, China) according to the manufacturer’s protocol. PCR amplification of the ITS-1 gene [33] was performed using *ApexHF* HS DNA Polymerase FS Master Mix (AG, China) with primers ITS-1 forward 5′-GATTTGCATTCAAGAAGCGTGATAGTAT-3′ and ITS-1 reverse 5′-AGTTTAGGAAGCAATCTGAAAGCACATC-3′ according to the manufacturer’s protocol. The first step of amplification was 3 min of denaturation at 95°C. This step was followed by 35 cycles, with 1 cycle consisting of 30 s at 55°C at the annealing temperature, and 30 s at 72°C. All PCRs were performed in a thermal cycler (Biometra, Germany). 6 μL of DNA loading buffer (AG, China) was added to the amplified PCR product, which was then separated by electrophoresis on a 1% agarose gel containing ethidium bromide and recorded using a digital gel documentation system (BioRad, USA).

### RNA-seq-method

The first step was the construction and sequencing of RNA extraction libraries. RNA was isolated and purified by Trizol method. Then the quantity and purity of total RNA was quality controlled with NanoDrop ND-1000 spectrophotometer (NanoDrop, United States). RNA integrity was then tested by Bioanalyzer 2100 (Agilent, United States) and also verified by agarose electrophoresis protocol. Using Dynabeads Oligo (dT) (Thermo Fisher, United States) magnetic beads, two rounds of purification were performed to specifically capture the mRNA with polyadenylate (Poly A) in it. The captured mRNA was fragmented under elevated temperature conditions (94°C for 5–7 min) using a Magnesium RNA Fragmentation Module (NEB, United States). Then the cleaved RNA fragments were reverse-transcribed to create the cDNA by SuperScript II Reverse Transcriptase (Invitrogen, United States), which were next used to synthesise U-labeled second-stranded DNAs with E. coli DNA polymerase I (NEB, United States), RNase H (NEB, United States) and dUTP Solution (Thermo Fisher, United States). An A-base was then added to the blunt ends of each strand, preparing them for ligation to the indexed adapters. Each adapter contained a T-base overhang for ligating the adapter to the A-tailed fragmented DNA. Dual-index adapters were ligated to the fragments, and size selection was performed with AMPureXP beads. After the heat-labile UDG enzyme (NEB, United States) treatment of the U-labeled second-stranded DNAs. The average insert size for the final cDNA librarys were 300±50 bp. Finally, 2×150 bp paired-end sequencing was performed using illumina Novaseq 6000 (LC-Bio Technology CO., Ltd., China) in PE150 sequencing mode according to the vendor’s recommended protocol. Fragments Per Kilobase of exon model per Million mapped reads (FPKM) was used to count the known genes in different samples. Differential gene expression analysis was performed in the hippocampus of infected and uninfected mice using DESeq2 software. The genes with the parameter of false discovery rate (FDR) below 0.05 and absolute fold change ≥ 2 were considered differentially expressed genes. Differentially expressed genes were then subjected to enrichment analysis of GO functions (http://www.geneontology.org/) [34] and KEGG pathways (https://www.kegg.jp/kegg/) [35].

### Hematoxylin and Eosin (H&E) staining

Mice half brains were fixed with 4% paraformaldehyde universal tissue fixative (Biosharp, China), dehydrated with gradient ethanol and clear xylene, and finally paraffin embedded. 6-μm-thick sagittal sections were made 2 mm to the left of the median brain and stained with H&E. Images were taken under a microscope (Leica, Germany).

### Western blot analysis

Tissue and cellular proteins were extracted using RIPA lysis buffer (Beyotime Biotechnology, China), and the lysates were centrifuged at 12000 rpm for 15 min at 4°C. The protein concentrations in the lysates were determined by the standard curve method using the BCA protein assay kit (Biosharp, China) with bovine serum albumin (BSA) as the standard and a microplate reader (Tecan, Switzerland). The protein samples were prepared by diluting the samples to the same concentration with RIPA lysis buffer, adding 1∕4 volume of SDS-PAGE Sample Loading Buffer (5×, Beyotime Biotechnology, China), and heating at 100°C for 10 min. The 30–60 μg protein samples were subjected to Sodium dodecyl sulfate-polyacrylamide gel electrophoresis (SDS-PAGE) and then transferred to polyvinylidene fluoride (PVDF) membrane using standard procedures. The membranes were closed with 5% skim milk dissolved in TBST (20 mM Tris-HCl pH 7.5, 150 mM NaCl, 0.1% Tween 20) for 2 h. After completion of closure, the membranes were washed three times with TBST for 10 min each. Rabbit polyclonal antibodies against GFAP, TGF-β, ARG1, TNF-α (1:1000, Affinity Bioscience, USA), IFN-γ, NRF2, COX2 (1:500, Wanleibio, China), TfR1, Fpn, NCOA4, SLC7A11, GPx4 (1:1000, Abmart, China), HO-1 (1:1000, HUABIO, China), rabbit monoclonal antibody against FTH1 (1:1000, HUABIO, China), and mouse monoclonal antibody against 4-Hydroxynonenal (1:1000, 4-HNE, R&D Systems, United States) were incubated with the PVDF membranes overnight at 4°C. Then washed PVDF membranes 3 times for 10 min each with TBST. The HRP-conjugated goat anti-rabbit/mouse IgG antibody (1:10000, Proteintech, China) was incubated for 1.5 h at room temperature and the membranes were washed 3 times for 10 min each with TBST. The protein bands were observed by an enhanced chemiluminescence detection system (BioRad, United States) and photographed.

### Inductively coupled plasma-mass spectrometry (ICP-MS) analysis

Tissue samples were washed 3 times with ultrapure water, blotted dry on filter paper and weighed precisely about 0.02 g of hippocampal tissue. Tissue samples were mixed with acid (HNO_3_:H_2_O_2_ = 2:1) in the DigiBlock Dissolver (LabTech, China) at 120°C three times, 1 mL each time with an interval of 10 min. Until the tissue samples became clear and clarified liquid, warmed up to 150°C to remove acid until the volume of liquid was less than 1 mL, with a constant volume to 10 mL using ultrapure water. The iron content was measured using Clin-ICP-QMS-I trace element meter (Bioyong Tech, China) with standard curve method. The elemental content of the sample was calculated according to the following equation. Hippocampal iron level = C V ∕ M, where C is the concentration of the measured element, V is the volume of the fixed volume of the sample, and M is the mass of the sample.

### Assay for glutathione (GSH)/glutathione disulfide (GSSG)

GSH and GSSG levels in tissues and cells were determined using the GSH and GSSG assay kits (Nanjingjiancheng, China) according to the manufacturer’s instructions. Approximately 30 mg of hippocampal tissue from each sample was prepared into 150 μL of 20% homogenate for the assay of GSH content, hippocampal GSH content was expressed as μmol/L. Each cell sample of approximately 3 × 10^7^ cells was prepared into 150 μL of homogenate for the assay, cellular GSH content was expressed as nmol/10^7^ cell.

### Assay for malonaldehyde (MDA)

Approximately 15 mg of hippocampal tissue was lysed into 10% tissue homogenate with PBS, and then the tissue lysate was centrifuged at 12,000 g for 10 min at 4°C, and the supernatant was collected for hippocampal MDA assay. Approximately 1 × 10^7^ cells were lysed by adding 150 μL of IP lysis buffer (Servicebio, China) and centrifuged at 12000 g for 5 min at 4°C, and the supernatant was collected for cellular MDA assay. MDA content was measured using a lipid peroxidation MDA assay kit (Nanjingjiancheng, China) following the manufacturer’s instructions.

### Transmission electron microscope observation of ultrastructure

The mice were weighed and anesthetized by intraperitoneal injection of 10% pentobarbital; the heart was perfused with PBS until the heart, liver, etc. faded to blood, and then perfused with 4% paraformaldehyde universal tissue fixative (Biosharp, China) until the mice were rigid throughout the body. The whole brain was taken, divided into left and right halves, and pre-fixed with Para-formaldehyde-glutaraldehyde in PBS (2%/2.5%, Macklin Biochemical, China) at room temperature for 30 min under protection from light, and the hippocampus of the mouse halves was bluntly peeled on ice. Tissue blocks of 1 mm^3^ size were taken from the hippocampus, placed in 1.5 mL EP tubes containing Para-formaldehyde-glutaraldehyde in PBS (2%/2.5%), continued to be fixed at 4°C under light-proof conditions, dehydrated and embedded, then sectioned with a Leica EM UC7 ultrathin sectioning machine (Leica, Germany), double stained with uranyl acetate-lead citrate, placed under a 120 kV transmission electron microscope (HITACHI, Japan) to observe the ultrastructural changes of the cells and photographed. For cell samples, the cells were first digested down with trypsin. Cells were transferred to a centrifuge tube, centrifuged at 1000 rpm for 3 min and the supernatant was discarded. Then, Para-formaldehyde-glutaraldehyde in PBS (2%/2.5%) was added to the cell precipitate, and the cells were pre-fixed at room temperature and protected from light for 30 min. The cell samples were further fixed at 4°C and protected from light, dehydrated and embedded, and then sectioned with a Leica EM UC7 ultrathin slicer, stained with uranyl acetate-lead citrate, and placed under a 120 kV transmission electron microscope to observe the cell ultrastructure. The cells were then sectioned with a Leica EM UC7 ultrathin section, stained with uranyl acetate and lead citrate, placed under a 120 kV transmission electron microscope to observe ultrastructural changes and photographed.

### Immunofluorescence detection of Transferrin receptor 1 (TfR1) and Ferritin heavy chain-1 (FTH1) expression

Paraffin-embedded mouse brain tissue was made into 6-μm-thick brain slices. The brain slices were baked at 60°C for 1 hour and then dewaxed sequentially in xylene and graded ethanol. Brain slices were rinsed three times with distilled water for 5 min each, and then placed in modified sodium citrate antigen repair solution (Biosharp, China) in a 95°C water bath for 20 min for antigen repair. Brain slices were washed three times with PBST (PBS + 0.1% Tween 20) for 5 min each, and then blocked with Immunol Staining Blocking Buffer (Beyotime Biotechnology, China) for 1 h at room temperature. The brain slices were washed three times with PBST for 5 min each, and then placed in a wet box and incubated with rabbit polyclonal antibodies TfR1 (1:100, Abmart, China) and rabbit monoclonal antibody FTH1 (1:200, HUABIO, China) at 4°C overnight. Brain slices were incubated at 37°C with iFluor 488 Conjugated Goat anti-rabbit IgG Goat Polyclonal Antibody (1:500, HUABIO, China) after three washes with PBST for 5 min each. Brain slices were washed three times with PBST and then added to Mounting Medium (antifading, with DAPI, Solarbio, China). Immunofluorescence images were obtained using a Leica DM6 B ortho-fluorescence Microscope (Leica, Germany). For immunofluorescence of cell samples, 3 immersions with PBS for 5 min each followed by blocking, incubation with TfR1 overnight and incubation with secondary antibody. All these steps were performed in 6-well plates, in the same way as for brain slices. After removing the cell crawl slices from the 6-well plate, added to Mounting Medium (antifading, with DAPI). Finally, observe and photograph by a Leica DM6 B ortho-fluorescence Microscope.

### Morris water maze (MWM)

The MWM performed in this study was adapted from the original study [36]. The experiment was started 1 day after infection. The experiments were conducted in a circular pool filled with water 1.2 m in diameter, made opaque with non-toxic paint and kept at a water temperature of 20–25°C. The maze was evenly divided into four quadrants and data were collected using the SMART 3.0 small animal behavioral recording and analysis system. Geometric patterns and colors were used as visual cues outside the maze. MWM was performed on 6 consecutive days; place navigation test was performed from day 1 to day 5, and spatial probe test was performed on day 6. Mice were transferred to the behavioral chamber the day before the start of training to acclimatize to the environment. During the training period, the platform was submerged 1 cm below the water surface in the center of the target quadrant and the mice were placed in a water maze facing the wall. Each mouse completed four 60 s trials per day, each with a randomized starting quadrant, and completed all quadrants in a clockwise direction. If a mouse failed to find the platform within the prescribed time frame, it was gently guided to the platform for 10 s. During the spatial probe test, the platform was removed and each mouse was placed in the quadrant opposite the target quadrant and allowed to search for the platform for 90 s.

### Reverse transcription and real-time PCR

Total RNA was isolated from TgCtwh3-infected mouse hippocampal tissue using the *SteadyPure* Quick RNA Extraction Kit (AG, China) according to the manufacturer’s protocol. RNA concentrations were determined using a micro UV spectrophotometer (NanoDrop, United States), and then all samples were reverse transcribed using a q-Script cDNA Synthesis Kit (Servicebio, China) according to the manufacturer’s protocols. Each sample contains 2 μg of total RNA. Quantitative PCR (qPCR) was performed using a SYBR Green Premix *Pro Taq* HS qPCR Kit (AG, China). Analysis of qPCR results based on the housekeeping gene GAPDH and calculation of relative gene expression levels by the Δ Δ Cq method [37]. The following primers were used in our study: GAPDH forward, 5′-GGTTGTCTCCTGCGACTTCA-3′; GAPDH reverse, 5′-TGGTCCAGGGTTTCTTACTCC-3′; *Tg*SAG1 forward, 5′-ACACGGCAGGCATCAAACTCAC-3′; *Tg*SAG1 reverse, 5′-CATACAACTCTGTGCGTCGTCTCC-3′.

### HT-22 cells culture and treatments

HT-22 cells were obtained from our laboratory and stored in liquid nitrogen tanks. HT-22 cells were cultured in Dulbecco’s modified Eagle’s medium (DMEM, Biological Industries, Israel) with 10% fetal bovine serum and 100 U/mL penicillin/streptomycin (37°C, 5% CO_2_). HT-22 cells as control without any treatment. The cells were pretreated with 100 μM DFP for 30 min before exposure to TgCtwh3 [21], HT-22 cells infected with TgCtwh3 tachyzoites (MOI = 3) for 24 h.

### Cell viability

Cell viability of HT-22 cells treated with 0, 25, 50, 75, 100, 125, 150 μM DFP for 24 h was measured using CCK-8 according to the manufacturer’s protocol (Biosharp, China).

### Cellular iron content assay

Iron assay was performed according to the manufacturer’s protocol of the Iron Colorimetric Assay Kit (Applygen, China). Briefly, the cells were collected, then washed twice with pre-cooled PBS, the PBS was aspirated and discarded, and 200 μL of lysis solution was added to lyse the cells for 2 h. The supernatant was collected as the specimens. The specimens were incubated with potassium permanganate solution at 60°C for 1 h followed by incubating for 30 min with an Iron ion detection agent at room temperature. The specimens were centrifuged at 12000 rpm for 5 min and the supernatant was used for the assay, then the microplate reader (OD = 550 nm) was used to detect the level of iron.

### Statistical analysis

GraphPad Prism 9.5.0 software was used for statistical analysis and graphing. Two-tailed Student’s t-test or Mann Whitney U test was applied to determine the statistical significance between two groups. One-way ANOVA or Brown-Forsythe and Welch’s ANOVA with Dunnett’s T3 multiple comparison test were applied for the multiple sets of data. Statistical differences were considered significant when *P*<0.05. All graphic data are presented as mean ± SD values.

## Results

### Observing brain damage and inflammation in mouse CT

**Fig 1:**
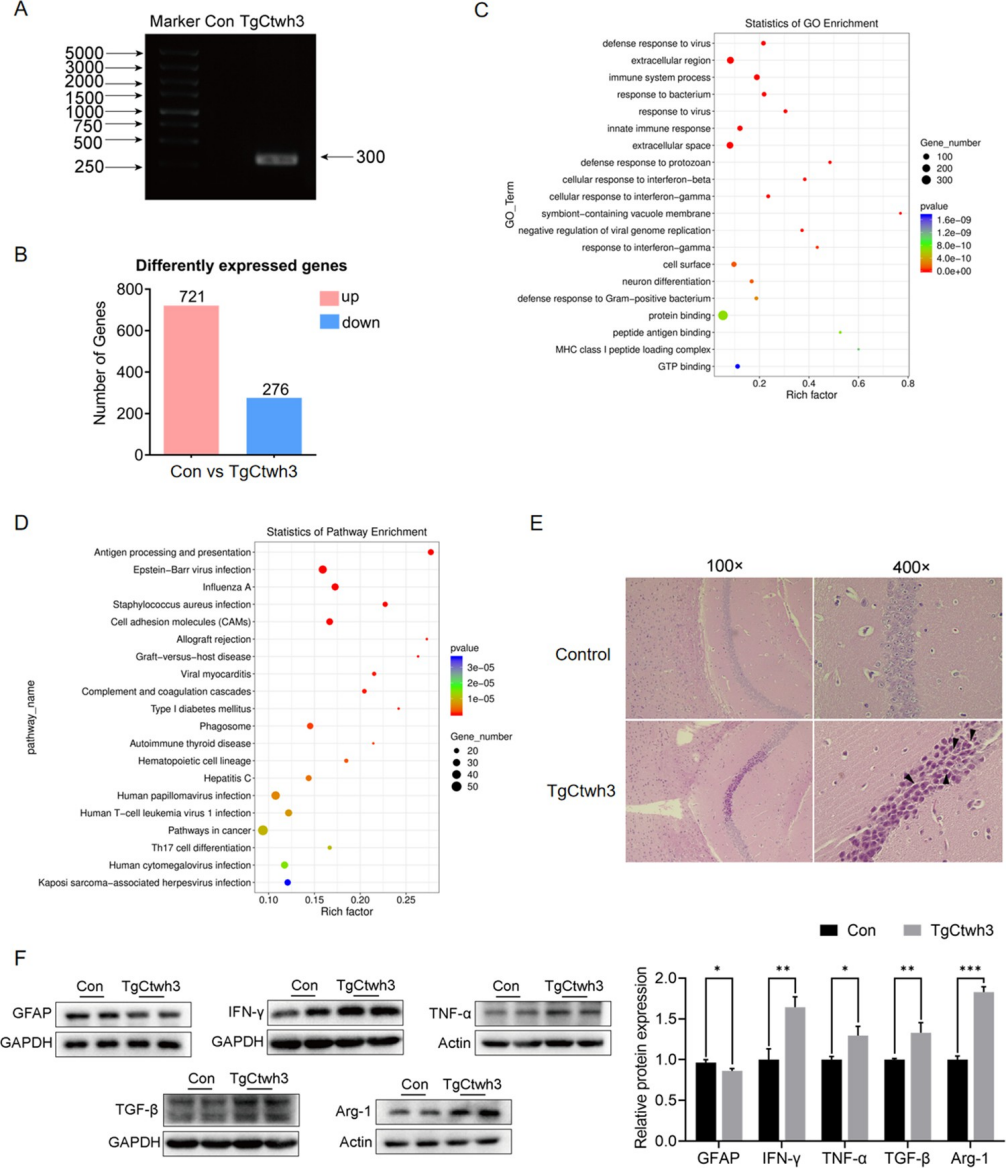
Construction and detection of the TgCtwh3 cerebral toxoplasmosis model. (A) Electrophoretic map of the hippocampal DNA of uninfected and infected mice. (B) RNA-seq data-based differentially expressed gene analysis between uninfected and infected mice hippocampus. Data of n = 3 biological replicates. (C) GO analyses of RNA-seq data showed the top 20 enriched GO terms pre- and post-infection. (D) KEGG analyses of RNA-seq data showed the top 20 enriched pathways in the hippocampus between pre- and post-infection. (E) Representative H&E images of hippocampal formation CA1 from uninfected and infected mice. Single black arrowheads, necrotic cells. (F) Western blot analysis of GFAP, IFN-γ, TNF-α, TGF-β and Arg-1 expression in hippocampus. *P<0.05, ** *P*<0.01, *** *P*<0.001. Mice were infected with TgCtwh3 for seven days.

C57BL/6 mice are one of the most commonly used mouse strains in neuro-science and are sensitive to *T*. *gondii* infection; the sensitive mouse strain C57BL/6 spontaneously develops necrotizing TE in the late stages of the infection [38]. Therefore, we used C57BL/6 mice to construct the TgCtwh3 CT model. ITS-1 was detected in the hippocampus after TgCtwh3 infection, indicating the presence of *T*. *gondii* in the hippocampus [33] (Fig 1A). An analysis of data obtained from gene sequencing in the hippocampus of pre- and post-infected mice identified 721 upregulated and 276 downregulated genes (Fig 1B). GO analyses of RNA-seq data showed that the top 20 enriched GO terms in the pre- and post-infection groups were mainly concentrated in the process of immune response, the antigen–worm reaction, and neuron differentiation (Fig 1C). KEGG analysis of the RNA-seq data showed that the top 20 pathways enriched before and after infection were mainly immune-related (Fig 1D). The HE results showed that the hippocampal formation CA1 appeared to have a wrinkled cytoplasm and the cell nuclei were deeply stained after TgCtwh3 infection (Fig 1E). In addition, the inflammatory cytokines Interferon-γ (IFN-γ), tumour necrosis factor α (TNF-α), transforming growth factor-β (TGF-β), and Arginase 1 (Arg-1) were significantly upregulated after the TgCtwh3 infection, while glial fibrillary acidic protein (GFAP) was down regulated (Fig 1F). *T*. *gondii* can cause necrotizing encephalitis in the non-immunocompromised host [39]. Damage to the CNS by *T*. *gondii* is characterized by many foci of enlarging necrosis and microglia nodules. The DNA of *T*. *gondii*, inflammatory factors, and necrosis were detected in the brains of mice infected for seven days, indicating that a mouse model of TgCtwh3 cerebral toxoplasmosis was successfully constructed and brain injury occurred.

### Ferroptosis occurred in the hippocampus of infected mice

**Fig 2:**
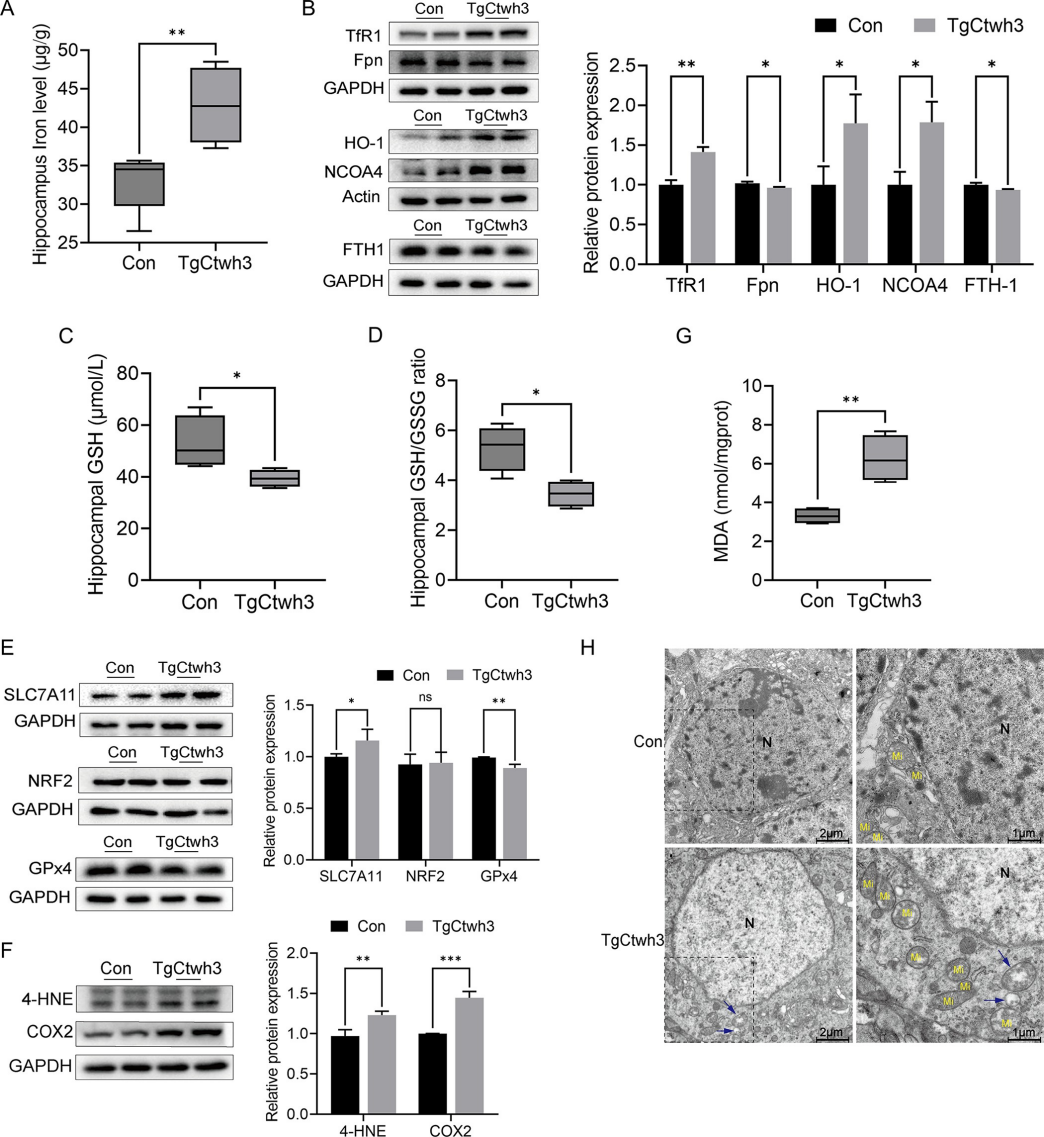
The expression of ferroptosis-related indicators and mitochondrial ultrastructural changes in the hippocampus of mice. (A) Determination of iron levels in the hippocampus of mice between uninfected and TgCtwh3 infected by ICP-MS. Data represent mean ± SD of n = 5 biological replicates. Statistical significance was calculated by two-tailed Student’s t-test. (B) Western blot analysis of TfR1, Fpn, HO-1, NCOA4, FTH1 expression in hippocampus. Hippocampal GSH (C) and GSH/GSSG ratio (D). Data represent mean ± SD of n = 4 biological replicates. Statistical significance of GSH was calculated by two-tailed Student’s t-test, and the data of GSH/GSSG ratio were analyzed using Mann Whitney U test. (E) Western blot analysis of SLC7A11, NRF2, GPx4 expression in hippocampus. (F) Western blot analysis of 4-HNE, COX2 expression in hippocampus. (G) MDA level in hippocampus. Data represent mean ± SD of n = 4 biological replicates. Statistical significance was calculated by Mann Whitney U test. (H) Electron micrographs showing mitochondria in hippocampus tissues obtained from uninfected and TgCtwh3 infected mice. N: nucleus; Mi: normal mitochondria; Blue arrow: damaged mitochondria. *P<0.05, ** *P*<0.01, *** *P*<0.001; ns, not significant. Mice were infected with TgCtwh3 for seven days.

Iron metabolism disorder and lipid peroxidation are the main characteristics of ferroptosis. Iron, the glutathione (GSH) level, and glutathione Peroxidase 4 (GPX4) function are closely related to ferroptosis sensitivity [40]. Our results showed that, seven days after the TgCtwh3 infection, the hippocampus iron augmentation was significantly higher than in uninfected mice (Fig 2A). Transferrin receptor 1 (TfR1), which mediates cellular iron uptake, was upregulated after the TgCtwh3 infection (Fig 2B). The membrane protein Ferroportin (Fpn/SLC40A1) responsible for intracellular iron efflux was downregulated (Fig 2B). Heme oxygenase-1 (HO-1) and nuclear receptor co-activator 4 (NCOA4) involved in converting bound iron into free iron were upregulated after TgCtwh3 infection (Fig 2B). Ferritin heavy chain-1 (FTH1) with iron oxidase activity was downregulated after infection (Fig 2B). The cyst(e)ine/GSH/ GPx4 axis plays a major role in ferroptosis resistance [41]. We found that TgCtwh3 infection led to a decrease in reduced GSH levels and the GSH/GSSG ratio in the hippocampus (Fig 2C and 2D). Meanwhile, GPx4 levels were downregulated (Fig 2E). Glutamate antiporter solute carrier family 7 member 11 (SLC7A11), one of the components of system Xc^-^, was upregulated (Fig 2E). There was no significant change in nuclear factor erythroid 2-related factor 2 (NRF2) protein levels before and after infection (Fig 2E). Additionally, 4-Hydroxynonenal (4-HNE) and cyclo-oxygenase-2 (COX2) were upregulated in the hippocampus of infected mice (Fig 2F). Malonaldehyde (MDA) is a lipid peroxidation product and was also elevated after TgCtwh3 infection (Fig 2G). Mitochondrial abnormalities could increase the sensitivity of ferroptosis [42]. Cells with normal nuclei but damaged mitochondria could be observed in our electron microscopic results (Fig 2H). To sum up, the iron overload induced ferroptosis in the hippocampus of TgCtwh3-infected mice.

### The effect of DFP on the expression of attenuated iron accumulation and lipid peroxidation in TgCtwh3-infected mice

**Fig 3:**
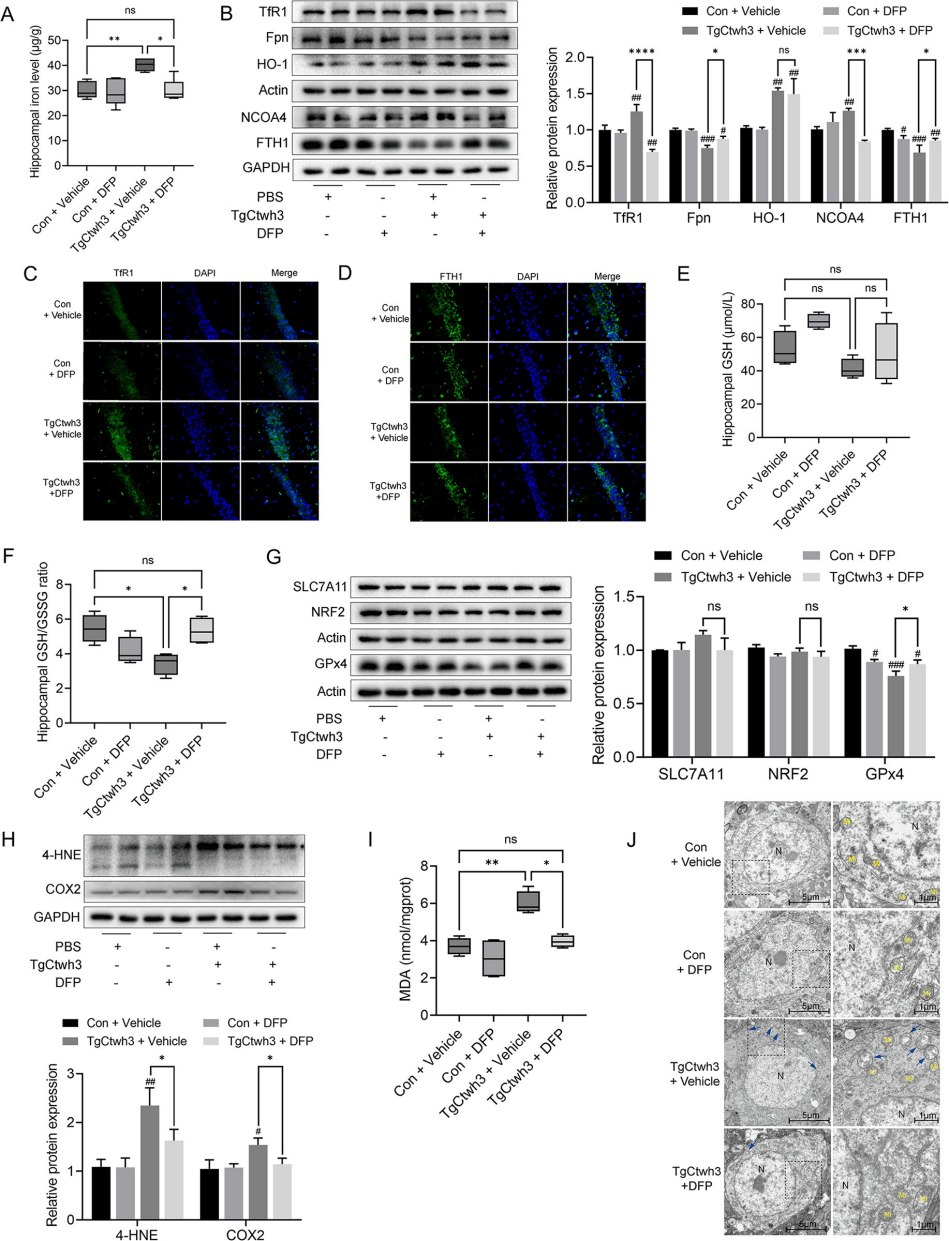
DFP inhibited ferroptosis and lipid peroxidation in the hippocampus of TgCtwh3-infected mice. (A) The iron levels in the hippocampus of mice in the con + vehicle, con + DFP, TgCtwh3 + vehicle and TgCtwh3 + DFP groups were determined using ICP-MS. Data represent mean ± SD of n = 5 biological replicates. (B) Western blot analysis of TfR1, Fpn, HO-1, NCOA4, FTH1 expression in the hippocampus. (C) Immunofluorescent images indicating TfR1 (green) in the CA1 region of the hippocampus at 400× with DAPI (blue). (D) Immunofluorescent images indicating FTH1 (green) in the CA1 region of the hippocampus at 400× with DAPI (blue). Hippocampal GSH (E) and GSH/GSSG ratio (F). Data represent mean ± SD of n = 4 biological replicates. (G) Western blot analysis of SLC7A11, NRF2, GPx4 expression in hippocampus. (H) Western blot analysis of 4-HNE, COX2 expression in hippocampus. (I) MDA level in hippocampus. Data represent mean ± SD of n = 4 biological replicates. (J) Electron micrographs of hippocampal tissue from uninfected mice, uninfected DFP-applied mice, TgCtwh3-infected mice, and TgCtwh3-infected DFP-applied mice. N, nucleus of TH-22 cells; Mi, normal mitochondria; blue single arrow, damaged mitochondria. Statistical significance was calculated by Brown-Forsythe and Welch’s ANOVA with Dunnett’s T3 multiple comparison test. #*P*<0.05, ##*P*< 0.01, ###*P*<0.001 versus control; *P<0.05, **P<0.01, ***P<0.001, ****P<0.0001; ns, not significant. Mice were infected with TgCtwh3 for seven days.

Following the DFP treatment, there was a significant iron level decrease in the hippocampus of TgCtwh3-infected mice compared with the control group (Fig 3A). Iron homeostasis in the hippocampus was also evaluated by measuring related proteins. TfR1 and NCOA4, which were upregulated after infection recovered to normal levels following DFP (Fig 3B and 3C). With the use of DFP after infection, Fpn and FTH1 slightly recovered, but they did not reach the levels observed in the control group (Fig 3B and 3D). HO-1, upregulated by infection with TgCtwh3, did not improve in response to DFP (Fig 3B). Then we detected GSH and GSSG in the hippocampus of the mice. GSH showed an increasing trend in the Con + DFP group, while it showed a decreasing trend after TgCtwh3 infection, but this result was not statistically significant (Fig 3E). However, DFP might reverses the decrease in the GSH/GSSG ratio in the hippocampus caused by TgCtwh3 infection (Fig 3F). No significant changes in SLC7A11 and NRF2 were observed in TgCtwh3 + DFP compared to the TgCtwh3 group (Fig 3G). There was a significant recovery in the GPx4 levels after the DFP treatment in the infected groups (Fig 3G). Moreover, 4-HNE, COX2, and MDA were reduced in the hippocampus of infected mice following the DFP treatment (Fig 3H and 3I). Combined with the reduction in mitochondrial damage in hippocampus after the use of DFP (Fig 3J), this result leads us to speculate that DFP could inhibit the iron accumulation and lipid peroxidation in the hippocampus of TgCtwh3 infected mice.

### DFP ameliorates CT by inhibiting ferroptosis and prolongs survival times in mice

**Fig 4:**
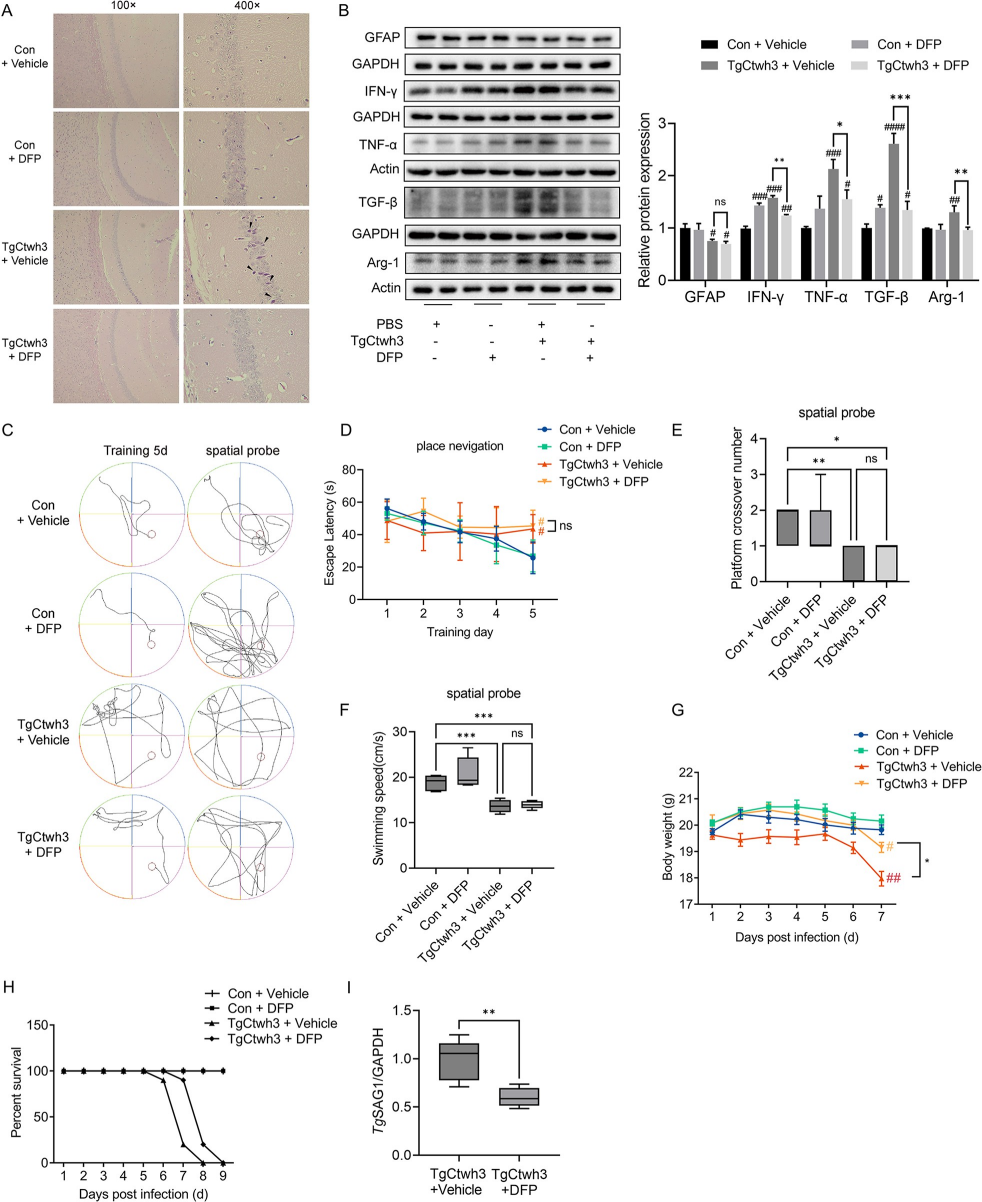
To detect the pathology, inflammation, behavior, survival, and *T*. *gondii* burden in mice after the use of DFP. (A) Representative H&E images of the hippocampal formation of CA1 from mice in the con + vehicle, con + DFP, TgCtwh3 + vehicle, and TgCtwh3 + DFP groups. Single black arrowheads, necrotic cells. (B) Western blot analysis of GFAP, IFN-γ, TNF-α, TGF-β and Arg-1 expression in hippocampus. (C) Morris water maze trajectory diagram of mice arriving at the platform on the fifth day of place navigation and the spatial probe test. (D) Escape latency of the place navigation. (E) Platform crossover number of the spatial probe. (F) Swimming speed of the spatial probe. (G) Body weight changes of mice in the above four groups. (H) Percent survival of mice in the above four groups, n = 10 mine. (I) QRT-PCR was used to detect the mRNA expression level of *Tg*SAG1 in hippocampus of TgCtwh3 infected mice, and GAPDH was used as reference mRNA, and statistical significance was calculated by two-tailed Student’s t-test, n = 6 mice. Data represent mean ± SD of n = 7 biological replicates. Statistical significance was calculated by Brown-Forsythe and Welch’s ANOVA with Dunnett’s T3 multiple comparison test. #*P*<0.05, ##*P*<0.01, ###*P*<0.001, ####*P*<0.0001 versus control; *P<0.05, **P<0.01, ***P<0.001; ns, not significant. Mice were infected with TgCtwh3 for seven days.

We found the iron-overload-induced ferroptosis in the hippocampus of TgCtwh3 infected mice. Furthermore, to explore the effect of DFP on the brain injuries of TgCtwh3-infected mice, we tested the pathological performance, cognitive ability, and survival of the mice. First, we found that DFP ameliorates TgCtwh3-infection-induced necrosis in CA1 regions of the hippocampus (Fig 4A). The protein expression levels of the inflammatory cytokines IFN-γ, TNF-α, TGF-β, and Arg-1 in the mouse hippocampus were reduced after DFP treatment for the TgCtwh3 infected mice (Fig 4B). However, DFP use could not improve the loss of GFAP (Fig 4B). Then, we performed the MWM experiment on four groups of mice. On the fifth day of the MWM, the uninfected mice had a shorter pathway to the platform than the infected mice (Fig 4C). In the spatial probe test, the uninfected mice stayed in the target quadrant for longer and crossed the platform more often than the infected mice (Fig 4C). In the first four days of the MWM, the times in which the mice from the four groups arrived at the platform did not show significant differences, but, on the fifth day, the infected group took longer to arrive at the platform than the control group (Fig 4D). Compared with the uninfected mice, the number of times the infected mice crossed the platform in the spatial probe test was significantly reduced, with no significant improvement after the use of DFP (Fig 4E). Additionally, the swimming speed of the infected mice was slower than that of the uninfected mice (Fig 4F). It seems that DFP does not improve the cognitive and motor functions of mice (Fig 4C-F). To observe the overall effect of DFP on the health of the mice, we recorded the changes in body weight of the mice at seven days post-infection. After the seventh day of infection, the body weight of the TgCtwh3-infected mice decreased significantly, and the use of DFP improved the body weight of the mice (Fig 4G). The use of DFP slightly prolonged the survival time of the mice infected with TgCtwh3 (Fig 4H). Finally, we found that the use of DFP decreased the expression of *Tg*SAG1 in the hippocampus, implying that DFP resulted in a reduction in the *T*. *gondii* burden in the hippocampus (Fig 4I).

### DFP prevented iron-overload-induced ferroptosis in HT-22 cells with TgCtwh3 infection

**Fig 5:**
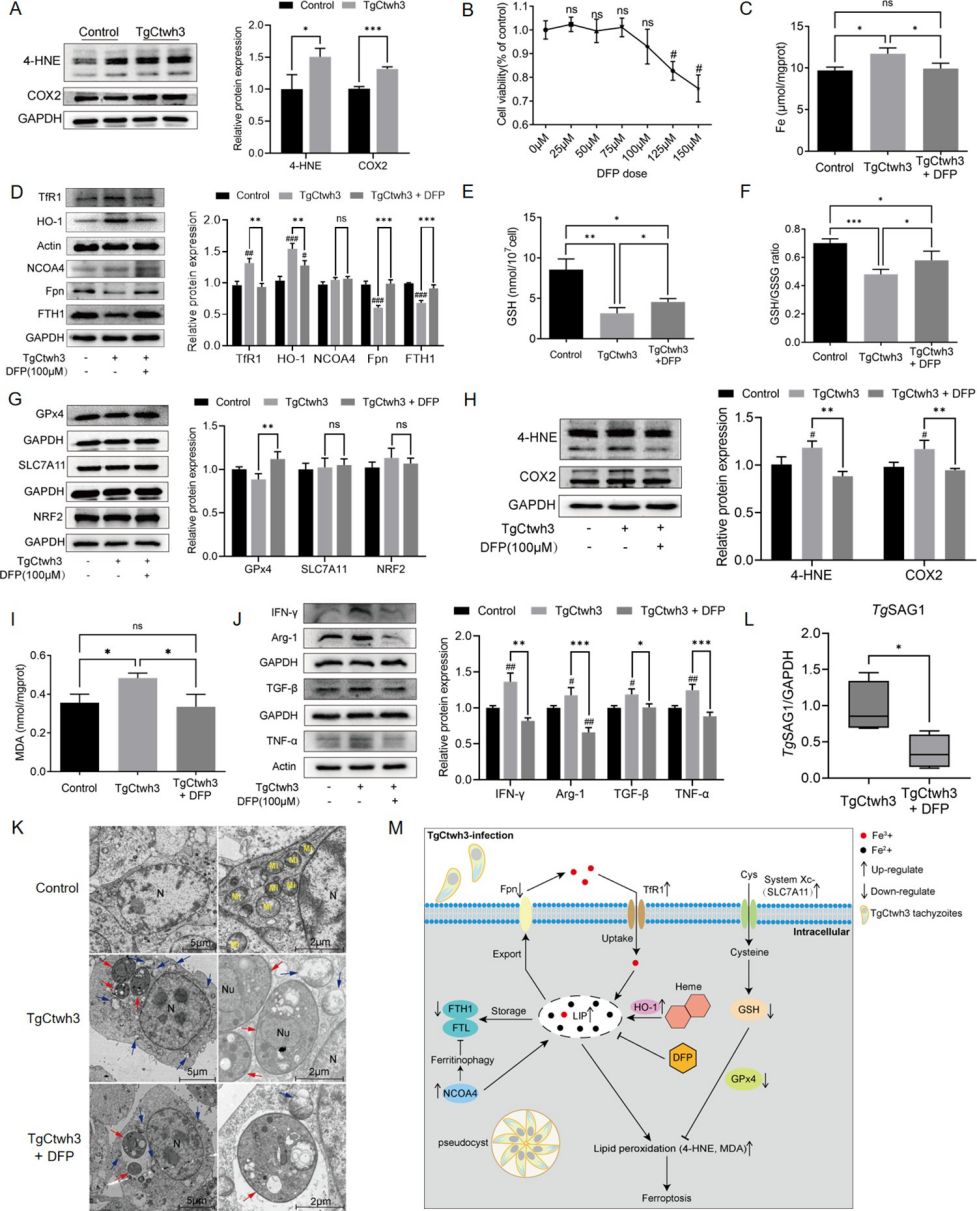
The iron-overload induced ferroptosis of TgCtwh3-infected HT-22 cells *in vitro* could be inhibited by DFP. (A) Western blot analysis of 4-HNE and COX2 expression in the HT-22 cells uninfected and infected with the TgCtwh3. (B) The CCK-8 test detects the cell viability of HT-22 cells after applying doses of 0, 25, 50, 75, 100, 125, and 150 μM of DFP. Data represent mean ± SD of n = 6 biological replicates. Statistical significance was calculated by Brown-Forsythe and Welch’s ANOVA with Dunnett’s T3 multiple comparison test. #*P*<0.05, ns, not significant versus 0μM DFP dose. (C) Iron was measured in the cells of control, TgCtwh3, and TgCtwh3 + DFP groups via an Intracellular Iron Colorimetric Assay. Data represent mean ± SD of n = 3 biological replicates. Statistical significance was calculated by one-way ANOVA. (D) Western blot analysis of TfR1, HO-1, NCOA4, Fpn, FTH1 expression in the above three groups of cells. GSH (E) and GSH/GSSG ratio (F) in the above three groups of cells. Data represent mean ± SD of n = 4 biological replicates. Statistical significance was calculated by one-way ANOVA. (G) Western blot analysis of GPx4, SLC7A11, NRF2 expression in the above three groups of cells. (H) Western blot analysis of 4-HNE, COX2 expression in the above three groups of cells. (I) MDA level of cells. Data represent mean ± SD of n = 4 biological replicates. Statistical significance was calculated by Brown-Forsythe and Welch’s ANOVA with Dunnett’s T3 multiple comparison test. (J) Western blot analysis of IFN-γ, TNF-α, TGF-β and Arg-1 expression of cells. (K) Transmission electron microscopy was used to observe the ultrastructure of the cells. N, nucleus of HT-22 cells; Nu, nucleus of TgCtwh3 tachyzoites; Red single arrow, TgCtwh3 tachyzoites; Mi, normal mitochondria; Blue single arrow, damaged mitochondria. (L) QRT-PCR was used to detect the mRNA expression level of *Tg*SAG1 in HT-22 cells, and GAPDH was used as a reference mRNA. Data of n = 4 biological replicates, and statistical significance was calculated by two-tailed Student’s t-test. (M) Schematic representation of the mechanism of action of ferroptosis in hippocampal cell damage due to TgCtwh3 infection. After the TgCtwh3 infection, LIP and overall cellular iron levels were elevated in response to TfR1, NCOA4, HO-1, Fpn, and FTH1, and excess iron promoted lipid peroxidation. GSH and GPx4 were downregulated after infection, and the antibody oxidation capacity of cells was decreased, which eventually led to lipid peroxidation and induced cellular ferroptosis. #*P*<0.05, ##*P*<0.01, ###*P*<0.001 versus control; *P<0.05, **P<0.01, ***P<0.001; ns, not significant. HT-22 cells were infected with TgCtwh3 for 24 h.

The expression of 4-HNE and COX2 was upregulated after the TgCtwh3 infection of HT-22 cells for 24 h (Fig 5A), suggesting that the TgCtwh3 infection of HT-22 cells for 24 h *in vitro* could lead to the occurrence of ferroptosis. In order to obtain a biosafe DFP concentrations, we performed a CCK-8 assay and showed that DFP concentration higher than 100 μM could inhibit cell growth, so we chose a DFP concentration of 100 μM for the *in vitro* experiments (Fig 5B). As iron homeostasis dysregulation is one of the main features of ferroptosis, we used an iron assay kit to detect the cellular iron levels. We found that TgCtwh3 infection could increase cellular iron levels and be improved by DFP (Fig 5C). Next, we detected protein expression related to iron metabolism using Western blot analysis, and the results showed that the expression of TfR1, HO-1, and NCOA4 was upregulated in HT-22 cells 24 h after TgCtwh3 infection. TfR1 and HO-1 protein expression levels to were equal to those in uninfected HT-22 cells, while NCOA4 protein expression levels were higher after the DFP treatment (Fig 5D). The protein expression levels of Fpn and FTH1 were downregulated in HT-22 cells infected with TgCtwh3, and DFP restored Fpn and FTH1 to normal levels (Fig 5D). DFP altered the GSS content and the GSH/GSSG ratio of the cells, which decreased after TgCtwh3 infection, but could not restore them to normal levels (Fig 5E and 5F). Meanwhile, we examined the expression of other components of the GPx4 axis using Western blot analysis, and the results showed that SLC7A11 and NRF2 did not change significantly in comparison to the control group. Meanwhile, GPx4 decreased after infection, and DFP could restore it to normal levels (Fig 5G). 4-HNE and MDA (Fig 5H and 5I) were both upregulated after TgCtwh3 infection, and DFP decreased 4-HNE and MDA levels. Our results showed that COX2 exhibited higher expression levels after TgCtwh3 infection, which DFP could inhibit. The above results show that HT-22 cells had characteristic indicators of ferroptosis after 24 h of TgCtwh3 infection, suggesting the occurrence of ferroptosis *in vitro*. DFP could improve or even eliminate these characteristic changes in infected cells. In addition to detecting the characteristics of ferroptosis, we examined cytokine transcripts, and the results showed that TgCtwh3-infected HT-22 cells exhibited higher levels of inflammatory factor expression, with significantly higher expression levels of IFN-γ, TNF-α, TGF-β and Arg-1 (Fig 5J). DFP reduced the levels of these inflammatory factors (Fig 5J). The electron microscopy results demonstrated that the mitochondrial structure was intact in HT-22 cells in their normal state. Almost all of the mitochondria in the TgCtwh3-infected HT-22 cells showed mitochondrial cristae breakage and disappearance or wrinkling. The mitochondria were less damaged and there were still intact mitochondria in the cells after the DFP treatment (Fig 5K). Compared to the TgCtwh3 group, the TgCtwh3 + DFP group had lower *Tg*SAG1 expression levels, indicating the effect of DFP on the *T*. *gondii* load (Fig 5L).

## Discussion

Ferroptosis has been shown to be involved in the mechanism of injury in mammalian CNS diseases such as Alzheimer’s disease [43,44], Huntington’s disease [45], Parkinson’s disease [25,46], and traumatic brain injury [47]. It is closely related the to cellular metabolism and is regulated by a variety of cellular metabolic events, including iron accumulation, redox homeostasis, and the mitochondrial, amino acid, lipid, and sugar metabolisms [48]. Ferroptosis can be induced by many mechanisms, which are mainly related to glutathione levels, GPx4 function, and the labile iron pool (LIP) [40]. Iron ions are usually bound to transferrin in the form of trivalent iron and enter the cell through the transferrin receptor. After the trivalent iron entering the cell is reduced to ferrous iron (Fe^2+^), various iron-binding complexes are preferentially formed to participate in various physiological and biochemical reactions. When the content of these complexes approaches saturation, excess divalent iron and very little trivalent iron accumulate in the cell, forming an LIP with redox activity [49]. TfR1, HO-1, NCOA4, FTH1 and Fpn are involved in the formation of LIP. One study found that *T*. *gondii* interfered with the expression of iron transporters and TfR1 to enhance iron internalization, favoring the growth of parasites [21]. We determined that TfR1 is upregulated after infection, which favors the internalization of iron and may favor the growth of *T*. *gondii*. HO-1 catalyzes the degradation of heme, releasing the bound iron in heme. *T*. *gondii* probably produces heme through its plant-like heme biosynthetic pathway, enhancing the acute virulence of *T*. *gondii* [50]. In our study, TgCtwh3 infection promoted the expression of HO-1 *in vivo* and *in vitro*, potentially increasing cellular free iron levels and favoring toxoplasma iron utilization. NCOA4 protein can convert bound iron into free iron for release into LIP through selective autophagy on ferritin [51]. FTH1, a component of ferritin, was downregulated in this study. The increase in NCOA4 may be the cause of the decrease in FTH1. Fpn synthesis is regulated by cellular hypoxia, iron and heme concentrations, and inflammatory signals. Fpn is involved in cellular iron redistribution, and Fpn downregulation leads to the impaired cellular iron efflux [52]. *In vitro* and *in vivo* studies have shown that TgCtwh3 infection can lead to downregulation of Fpn, which is not conducive to cellular iron excretion. After infection with TgCtwh3, the TfR1 responsible for iron intake in the cells is decreased; the bound iron stored in FTH1 and heme is released, respectively, by NCOA4 and HO-1, Additionally, the Fpn responsible for iron exclusion in the cells is downregulated. This leads to increased LIP levels in the cells and increased total iron levels in the hippocampus. Increased LIP is more involved in oxidative stress and the Fenton and Haber–Weiss reactions, which may lead to an increased membrane structure and DNA damage in cells. DFP chelates trivalent iron [24], reducing LIP participation in the reaction, thereby protecting the cell.

Astrocytes play a role in supporting and separating nerve cells and are involved in the formation of the blood–brain barrier, a barrier that *T*. *gondii* needs to break through at an early stage to enter the brain tissue [53]. Astrocytes can take up the glutamate and gamma-aminobutyric acid released by neurons, which can release glutamine after it is catalyzed by enzymes [54]. GFAP is expressed mainly in astrocytes; it is an important component of the cytoskeleton during astrocyte development and a marker of glial cell activation, and it is involved in the astrocyte response to CNS injury [55]. One study found that C57BL/6 mice that were deficient in GFAP experienced impaired inflammatory lesions and parasite control, ultimately leading to fatal necrotizing TE [56]. It has been shown that chronic infection with ME49 envelopes induces morphological and molecular changes in chronic astrocytes of C57BL/6 mice, leading to increased extracellular glutamate concentrations [57]. The results of our *in vivo* experiments show that GFAP expression was downregulated whereas SLC7A11 was upregulated after TgCtwh3 infection. The downregulation of GFAP expression indicates that astrocytes have impaired functions in TgCtwh3-induced CT, with impairments of the glutamate metabolism, neurotransmitter delivery, and the blood–brain barrier, which are ultimately detrimental to the astrocyte response to CNS injury. Impaired astrocytes result in higher extracellular glutamate levels and attenuate abilities related to cellular protection from glutamate neurotoxicity. The function of SLC7A11 is to import extracellular cystine in exchange for intracellular glutamate [58]. The upregulation of SLC7A11 exacerbates the export of cellular glutamate after TgCtwh3 infection *in vivo*. In contrast, in *in vitro* experiments, perhaps due to the absence of glutamate deficiency stress on the cells, there were no significant changes in SLC7A11. The insufficient glutamine uptake by neuronal cells due to insufficient glutamine conversion by astrocytes ultimately leads to a complete imbalance in the cellular glutamate cycle and the partial depletion of intracellular glutamate available for the tricarboxylic acid cycle. All of the above factors result in accelerated cell death. GSH is composed of glutamate, cysteine and glycine, which are involved in antioxidant activity and the detoxification of cells and are also key substances in the fight against ferroptosis. The disruption of the glutamate cycle is also a disruption of GSH synthesis. Our results showed that TgCtwh3 infection impaired the cystine/GSH/GPx4 axis, an effect that could be relieved by DFP. NRF2 regulates HO-1 and promotes the utilization and regeneration of GSH synthesis [59]. There was no significant difference in the changes in NRF2 after TgCtwh3 infection *in vivo* and *in vitro*, meaning that the limited rescue of GSH depletion was not facilitated. DFP had no regulatory effect on NRF2 expression. Levels of 4-NNE and MDA (two indicators of lipid peroxidation) were increased in the hippocampus and HT-22 cells due to the damage of the antioxidant axis caused by TgCtwh3 infection; they could be attenuated by DFP. All the above results are identical to the metabolic and physiological indicators related to ferroptosis and combine with the damage to mitochondria caused by TgCtwh3 infection, especially in cells containing *T*. *gondii* tachyzoites *in vivo*.

During the acute phase of CT, T cells and NK cells produce IFN-γ and TNF-α. Monocytes and M1 microglia produce large amounts of TNF-α, inducible nitrogen monoxide synthase (iNOS), and reactive oxygen intermediates, which help control the parasite burden in the host, but excessive inflammation and neuronal loss can also cause the host to eventually die of toxoplasma encephalitis [2]. We found that TgCtwh3 infection led to hippocampal damage, producing large amounts of pro-inflammatory cytokines, including IFN-γ and TNF-α. The overproduction of pro-inflammatory cytokines may lead to hippocampal-damage-related injuries. *T*. *gondii* can evade the immune response indirectly by increasing the expression of anti-inflammatory cytokines including TGF-β [60]. Therefore, TGF-β can be considered an important factor for toxoplasmosis. In the present study, TGF-β was upregulated after infection, and it may be regulated by oxidative stress [61] and *T*. *gondii* [60]. Meanwhile, we found that Arg-1 expression was upregulated in the hippocampus and HT-22 cells of mice after TgCtwh3 infection and that it decreased in response to DFP. *T*. *gondii* can use Arg-1 from the host to inhibit NO production and thus protect itself [62]. Then, the inhibition of Arg-1 by DFP seems to inhibit the immune regulation of *T*. *gondii* against the host. Ferroptosis can contribute to the development of inflammation and participate in the disease process via several pathways [23]. DFP inhibited the occurrence of ferroptosis in the hippocampus. After ferroptosis was inhibited, inflammatory factors in the hippocampus of the infected mouse were similarly inhibited, suggesting that ferroptosis an important role in TE. Despite the improved inflammation and pathology in the hippocampus, the swimming speed and cognitive performance of the mice did not improve, perhaps due to *T*. *gondii* acting via the non-ferroptosis pathway. Despite the control of the ferroptosis and the reduced parasite burden, DFP produced only one extra day of survival. *T*. *gondii* infection leads to an increase in the host’s iron levels. Additionally, the DFP drug used in this study did indeed improve the host’s overall iron levels, leaving the host with a negative iron balance, which is not conducive to the availability of *T*. *gondii* to iron. Some studies have shown that the *T*. *gondii* vacuolar iron transporter plays an important role in coping with acute iron deficiency and iron excess [63]. The strain used in this study is a wild-type strain. As far as *T*. *gondii* itself is concerned, it has no defects terms of in iron transport and resistance to iron deficiency. It is not clear what kind of *T*. *gondii* effectors play a role in the process whereby *T*. *gondii* competes for iron with its host, but this process may be limited by DFP, which limits the speed with which *T*. *gondii* obtains iron in a single cell. Controlling the iron levels in a cell can prevent the ferroptosis of cells, which may prevent *T*. *gondii* from releasing and spreading prematurely. Although ferroptosis was inhibited in our study, the GFAP expression levels were not improved; moreover, the *Tg*SAG1 gene of *T*. *gondii* was still detected in the TgCtwh3 + DFP group, indicating that the processes of *T*. *gondii* replication and active host cell lysis could not be completely controlled.

DFP is a multi-target drug approved by the European Medicines Agency and the Food and Drug Administration; it has a strong affinity for iron [64], binding iron in a 3:1 (DFP: iron) molar ratio and increasing urinary iron excretion. DFP is used as a clinical therapy worldwide in thalassemia and other non-iron-loading disorders, such as Friedreich’s ataxia [65], pantothenic kinase-associated neurodegeneration [66], Parkinson’s disease [67] and other similar cases. The modalities of action of DFP include iron chelation [24], antioxidant [68], anti-hypoxic [69], and anti-ferroptosis effects, and iron mobilization in immune cells and cells involved in hyperinflammation [70], etc. The results of this study show that the iron chelator DFP exerts antioxidant effects by blocking iron accumulation, improving the expression of cellular iron-metabolism-related proteins (TfR1, HO-1, NCOA4, FTH1, Fpn), ultimately resisting cellular ferroptosis, and improving the pathological manifestation and inflammation of the hippocampus as well as the overall health of the mice, suggesting the possibility of treating CT with DFP.

In the present study, we assessed the role of ferroptosis in CT mice infected with virulent TgCtwh3. We found that TgCtwh3-induced cell death in the models used is dependent on GSH, GPx4 levels, unstable iron accumulation, and lipid peroxidation, as is consistent with the main criteria for ferroptosis (Fig 5M). Furthermore, we further showed that the blocking of iron accumulation by the iron chelator DFP inhibited ferroptosis and improved the pathological manifestations and inflammation of the hippocampus as well as the overall health of the mice, suggesting the possibility of using DFP to resist cellular ferroptosis in the treatment of CT.
